# Pax6 Interactions with Chromatin and Identification of Its Novel Direct Target Genes in Lens and Forebrain

**DOI:** 10.1371/journal.pone.0054507

**Published:** 2013-01-14

**Authors:** Qing Xie, Ying Yang, Jie Huang, Jovica Ninkovic, Tessa Walcher, Louise Wolf, Ariel Vitenzon, Deyou Zheng, Magdalena Götz, David C. Beebe, Jiri Zavadil, Ales Cvekl

**Affiliations:** 1 The Department of Genetics, Albert Einstein College of Medicine, Bronx, New York, United States of America; 2 The Department of Ophthalmology and Visual Sciences, Albert Einstein College of Medicine, Bronx, New York, United States of America; 3 The Department of Neurology, Albert Einstein College of Medicine, Bronx, New York, United States of America; 4 Department of Ophthalmology and Visual Sciences, Washington University School of Medicine, St. Louis, Missouri, United States of America; 5 Helmholtz Zentrum Munchen, Institute of Stem Cell Research, Ingolstädter Landstraße 1, Neuherberg, Germany; 6 Institute of Physiology, Department of Physiological Genomics, Ludwig-Maximilians-University Munich, Munich Center for Integrated Protein Science CiPS, Munich, Germany; 7 Department of Pathology, New York University Langone Medical Center, New York, New York, United States of America; The Walter and Eliza Hall of Medical Research, Australia

## Abstract

Pax6 encodes a specific DNA-binding transcription factor that regulates the development of multiple organs, including the eye, brain and pancreas. Previous studies have shown that Pax6 regulates the entire process of ocular lens development. In the developing forebrain, Pax6 is expressed in ventricular zone precursor cells and in specific populations of neurons; absence of Pax6 results in disrupted cell proliferation and cell fate specification in telencephalon. In the pancreas, Pax6 is essential for the differentiation of α-, β- and δ-islet cells. To elucidate molecular roles of Pax6, chromatin immunoprecipitation experiments combined with high-density oligonucleotide array hybridizations (ChIP-chip) were performed using three distinct sources of chromatin (lens, forebrain and β-cells). ChIP-chip studies, performed as biological triplicates, identified a total of 5,260 promoters occupied by Pax6. 1,001 (133) of these promoter regions were shared between at least two (three) distinct chromatin sources, respectively. In lens chromatin, 2,335 promoters were bound by Pax6. RNA expression profiling from Pax6^+/−^ lenses combined with *in vivo* Pax6-binding data yielded 76 putative Pax6-direct targets, including the Gaa, Isl1, Kif1b, Mtmr2, Pcsk1n, and Snca genes. RNA and ChIP data were validated for all these genes. In lens cells, reporter assays established Kib1b and Snca as Pax6 activated and repressed genes, respectively. In situ hybridization revealed reduced expression of these genes in E14 cerebral cortex. Moreover, we examined differentially expressed transcripts between E9.5 wild type and Pax6^−/−^ lens placodes that suggested Efnb2, Fat4, Has2, Nav1, and Trpm3 as novel Pax6-direct targets. Collectively, the present studies, through the identification of Pax6-direct target genes, provide novel insights into the molecular mechanisms of Pax6 gene control during mouse embryonic development. In addition, the present data demonstrate that Pax6 interacts preferentially with promoter regions in a tissue-specific fashion. Nevertheless, nearly 20% of the regions identified are accessible to Pax6 in multiple tissues.

## Background

A fundamental feature of gene regulation during embryonic development is an extensive utilization of combinatorial mechanisms that ensure proper temporal and spatial control of gene expression [1]. The majority of transcription factors that regulate embryonic development are expressed in multiple tissues [2]. The combinatorial regulatory mechanism at the “ground” level employs specific combinations of lineage-restricted sequence-specific DNA-binding transcription factors to control cell-specific gene regulatory networks [3], [4], [5]. The emergence of ChIP-chip and ChIP-seq technologies to identify regions of chromatin occupied *in vivo* by specific transcription factors, to map local chromatin structure, and to assess long-range interactions between specific regions of chromatin allows novel insights into the process of gene regulation [6].

Pax6 (paired box 6) is a lineage-specific DNA-binding transcription factor that regulates development of the central nervous system, endocrine pancreas, eye and olfactory system [7], [8], [9], [10], [11], [12], [13]. A series of studies implicated Pax6 and its homologues (e.g. *Drosophila eyeless* (*ey*) and *eye gone* (*Eyg*), zebrafish Pax6a and Pax6b) and related (jellyfish Pax-A and Pax-B) genes as central molecules responsible for the evolution of visual systems in vertebrates and invertebrates [14], [15], [16], [17]. Consequently, a number of mouse and human developmental defects and neurological disorders have been attributed to mutations in Pax6/PAX6 genes [18], [19]. Thus, understanding of genetic networks downstream of Pax6 genes is essential for better understanding of embryonic development and mechanism of human congenital diseases.

In the lens, Pax6 serves as an essential gene for the formation of lens progenitor cells. In the absence of Pax6, the lens placode is not formed [20], [21]. In subsequent stages, Pax6 regulates lens morphogenesis during lens vesicle formation [22], [23], [24], the withdrawal of primary lens fiber cells from the cell cycle and their terminal differentiation [25], [26], [27], [28], [29]. In the developing brain, Pax6 controls neurogenesis, patterning and proliferation in neuroepithelial and radial glia progenitor cells [12]. In addition, Pax6 is required for adult neurogenesis, and dopaminergic olfactory bulb interneuron specification and survival [30], [31], [32]. In the pancreas, Pax6 controls the formation of islets, the number of endocrine cells, in particular α- and β- cells. Pax6 directly regulates key functional genes, including glucagon, glucose transporter 2, insulin and somatostatin [33], [34], [35], [36], [37], [38]. Recent comparative RNA profiling studies showed that Pax6 regulates expression of selected common genes in lens and in embryonic forebrain [26]. Specifically, Pax6 directly controls expression of αA-crystallin in lens [39] and in olfactory bulb dopaminergic neurons [32] via shared but not identical molecular mechanisms. Thus, these findings raise the interesting possibility that some Pax6-target genes are common between multiple tissues while other targets are tissue-specific.

Considering the central role of Pax6 in eye formation, it has been estimated that *Drosophila ey* ultimately controls the expression of several thousand genes [40], [41], [42]. However, the proportion of directly or indirectly regulated genes is not known, as the majority of Pax6-direct targets in different tissues and at specific developmental stages remain to be determined [26], [43], [44]. To begin answering these questions, we performed comparative chromatin immunoprecipitation (ChIP) studies with Pax6-specific antibodies using chromatin prepared from newborn mouse lens, E15 forebrain and cultured pancreatic β-cells. To identify genes regulated by Pax6, we performed analysis of transcripts expressed in the wild type lens placode and compared them with genes expressed in Pax6^−/−^ surface ectoderm. In addition, we used earlier published data on differential gene expression between wild type and Pax6^+/−^ lenses [26] as well as wild type and Pax6^−/−^ embryonic cortex [26], [45]. These studies identified a series of novel Pax6 direct target genes, with Kif1b and Snca characterized at both the cellular and molecular levels.

## Results

### Identification of Pax6-bound regions in lens, forebrain and β-cell chromatin

**Figure 1:**
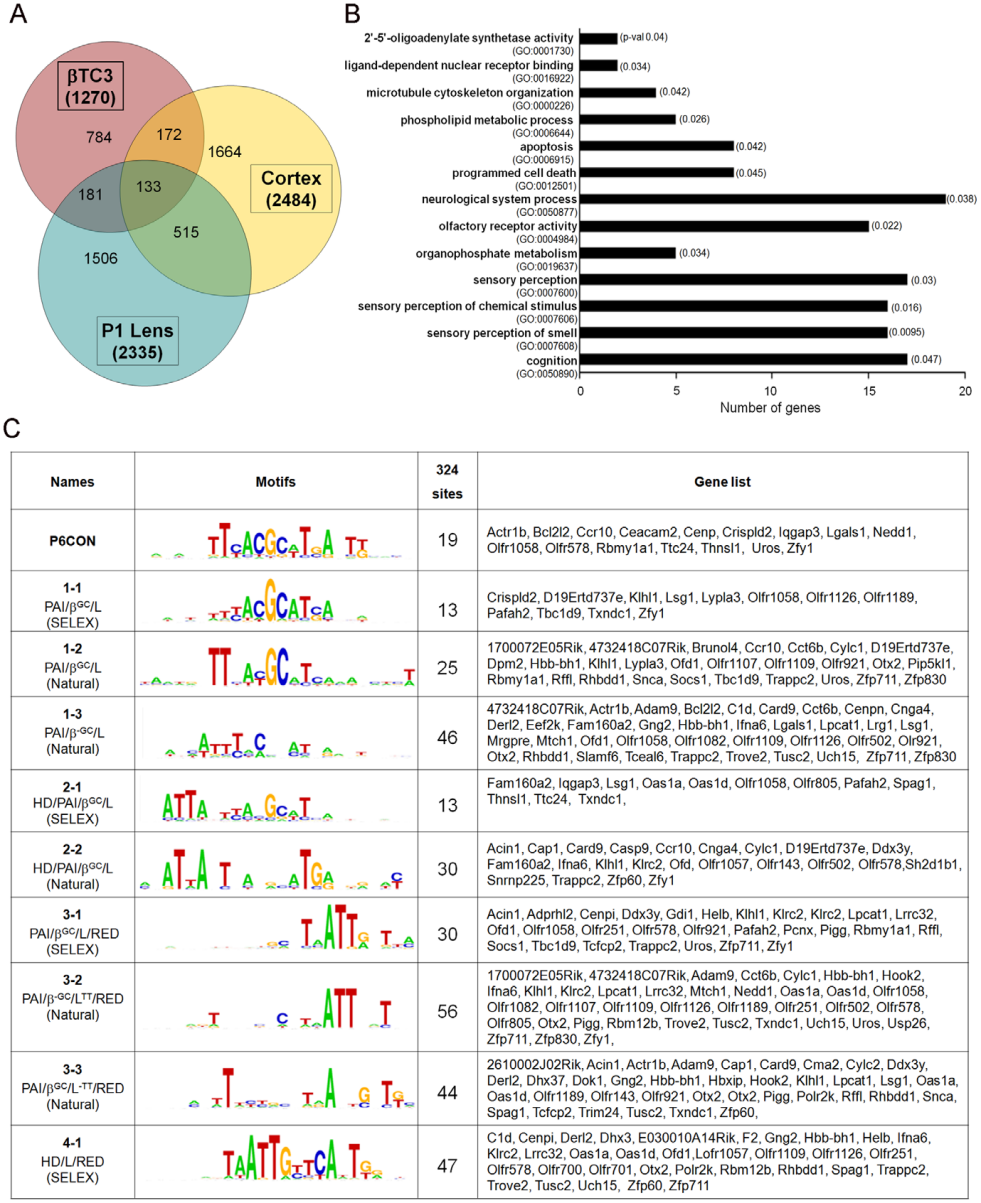
Pax6-occupied peaks in chromatin prepared from lens, cortex and pancreatic β-cells. (A) Venn diagram showing number of peaks occupied by Pax6 in lens, cortex and β-cell chromatin. (B) The gene ontology (GO) classification of 133 genes that are occupied by Pax6 in all three chromatins. (C) Analysis of distribution of ten novel Pax6-binding site motifs in Pax6 peaks at the 133 gene loci, through FIMO (Find Individual Motif Occurrences) predictive tool.

We reasoned that parallel studies of multiple tissues/cells regulated by Pax6, such as lens, forebrain and cultured β-cells of pancreas, would increase our power to identify tissue-specific as well as shared Pax6-dependent regulatory pathways. To identify regions occupied by Pax6 in chromatin prepared from newborn lenses, E15 forebrain and pancreatic β-cells, we used GeneChip Mouse Promoter 1.0R Arrays (Affymetrix), which contain over 28,000 mouse promoters. Each promoter region covers approximately 6 kb upstream and 2.5 kb downstream of the transcription start site(s). Three biological replicates were performed using each tissue. The data were analyzed using Model-based Analysis of Tiling-arrays [46] using a MAT score of 4.0 as the cutoff. We found a total of 5,260 regulatory elements occupied by Pax6. In individual materials, we found 2,335, 2,484 and 1,270 Pax6-bound promoters in lens, forebrain and β-cells chromatins, respectively (Figure 1 and Table S1). The Venn diagram (Figure 1A) shows that a total number of 133 genes had their promoter regions bound by Pax6 in the three tissues. Commonly occupied promoter regions were also found between the lens and cortex (n = 515), lens and β-cells (n = 181) and cortex and β-cells (n = 172). The total number of genes/promoters occupied by Pax6 in at least two tissues was 1,001, representing ∼20% of all genes/promoters bound by Pax6.

To further characterize these 133 genes, we investigated their ontology and presence of the Pax6 DNA-binding motifs within the bound regions. The largest GO categories, olfactory receptor activity, sensory perception of chemical stimulus and sensory perception of smell, include 15 genes encoding olfactory receptors (Figure 1B). Interestingly, only four from the set of 133 genes were previously identified as Pax6-regulated genes: *Gaa*, *Nav1*, *Otx2* and *Snca* [26], [45], [47]. To identify the putative Pax6-binding sites, we employed a recently established set of ten Pax6-binding site motifs [48]. These DNA motifs represent a continuum of related sequences presumably recognized by different combinations of the Pax6 protein structural motifs identified via crystallographic studies. These include the β-turn, PAI, linker, RED and homeodomain (HD) [49], [50]. These DNA motifs were converted into position-specific scoring matrices using the Python program and the potential binding sites were found by a software prediction tool, Find Individual Motif Occurrences (FIMO) [51]. A total of 324 unique binding sites were identified (Figure 1C). Each of these individual motifs predicted a comparable number of putative Pax6-binding sites (median 30, range 13 to 56) in approximately 80% of the “peak” regions examined (Figure 1C).

### Identification of direct Pax6 target genes in lens development

**Figure 2:**
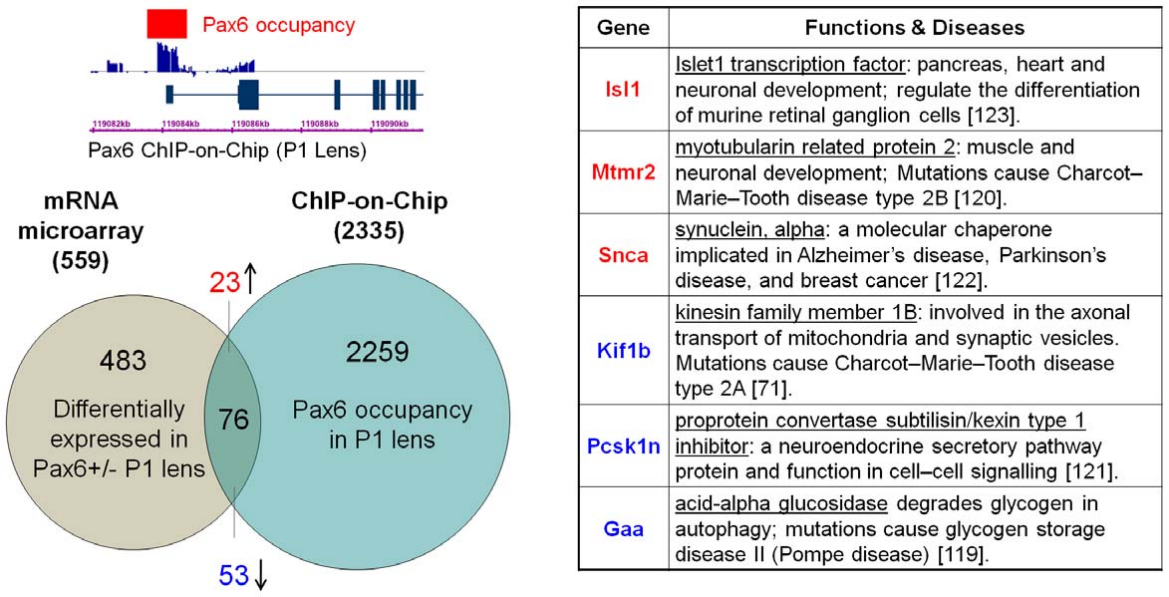
Identification of genes regulated by Pax6 in newborn lens. Venn diagram showing identification of 76 genes both bound and regulated by Pax6 by intersecting two genome-wide data sets: RNA expression profiling (559 transcripts, P1 lens, Pax6^+/−^ versus wild type) [26] and present ChIP-chip studies (2,335 peaks) (Table S1) in lens chromatin. The table summarized the known function of six important and validated Pax6 direct target genes from this group of 76 genes (see Figure 4) [75], [121], [122], [123], [124], [125].

To find candidate genes for direct regulation by Pax6 in lens, we first compared the 2,335 Pax6-peaks identified in newborn lens chromatin (Table S1) with 559 genes differentially expressed in Pax6 heterozygous lenses (accession number GSE13244) of the same developmental stage identified earlier [26]. This analysis revealed 53 down- and 23 up-regulated genes (Figure 2). The relatively low number of overlapping genes (n = 76) could be explained by the fact that the RNA expression profiling identified genes sensitive only to Pax6 haploinsufficiency.

**Figure 3:**
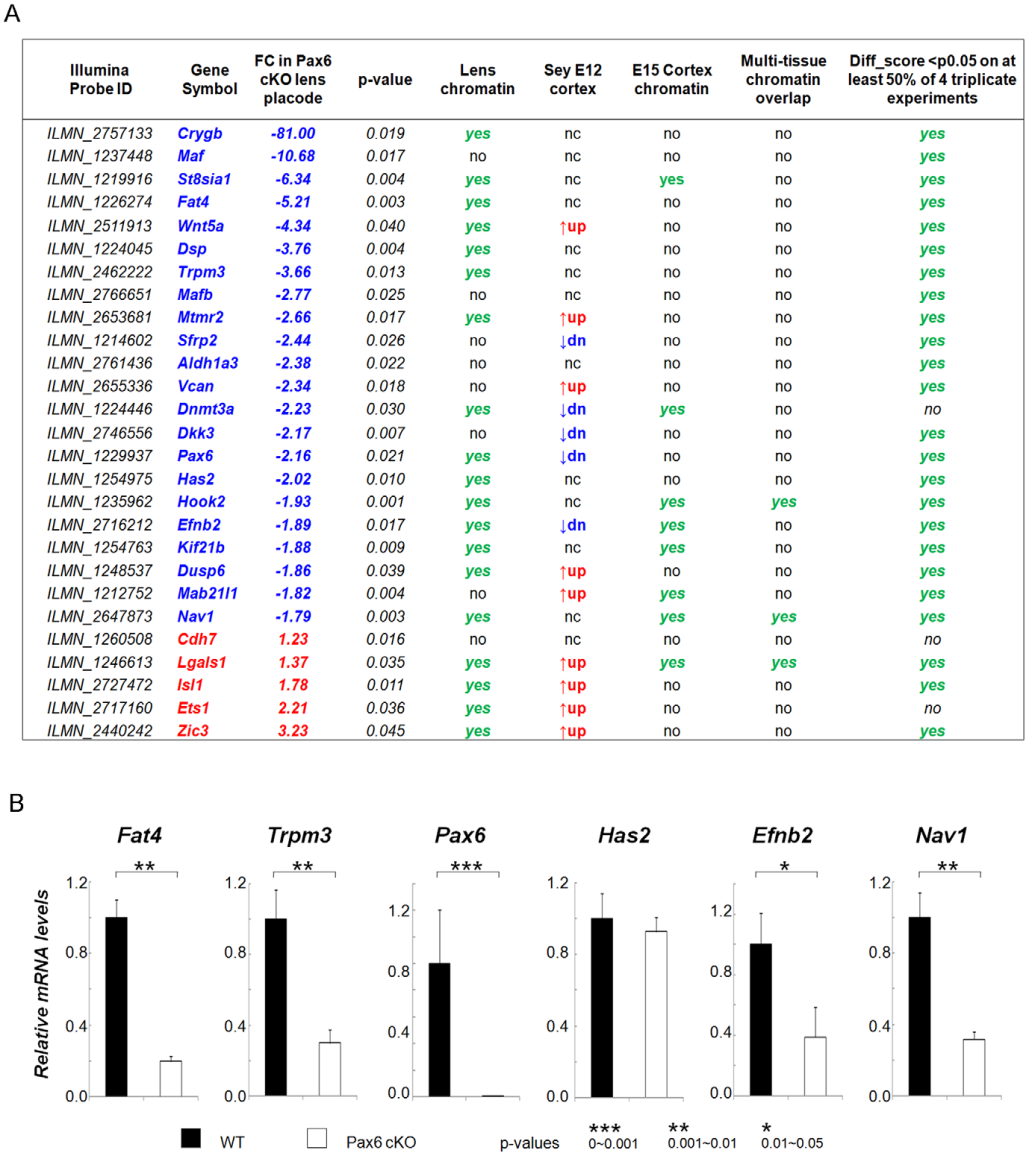
Twenty-seven genes relevant to lens placode formation and lens morphogenesis show differential expression in Pax6^−/−^ E9.5 mutated lens placodes. (A) A list of 27 genes includes a combination of well-characterized genes in lens biology and selected differentially expressed genes in Pax6 null (*Sey*) cortex [45]. The differentially expressed genes in Pax6^−/−^ E9.5 wild type and mutated lens placodes were identified using the Illumina Mouse6 bead microarrays as described elsewhere [96]. Twenty-four of the 27 genes were differentially expressed in at least 50% of experiments. (B) Relative expression levels of *Fat4*, *Trpm3*, *Pax6*, *Has2*, *Efnb2*, and *Nav1* in wild type (WT, black bars) and Pax6^−/−^ (open bars) lens placode and mutated ectoderm were determined using qRT-PCR as described in Methods.

To identify genes whose expression is dependent on both copies of Pax6, we performed conditional inactivation of Pax6 in the lens placode using the Le-Cre system [20]. In Pax6 null embryos, lens placodes are not formed [52]. Using the laser microdissection in combination with RNA expression profiling detected by Illumina Mouse WG-6 V1 1 R4 expression BeadChips, we identified several hundreds candidates of differentially expressed transcripts between the wild type lens placode and Pax6^−/−^ surface ectoderm (E9.5) as described in Materials and Methods. Figure 3A shows a list of 27 candidate genes that were selected according to their established or prospective roles in lens placode morphogenesis, and identified as differentially expressed in Pax6 null embryonic cortex [45]. Identification of Pax6 “peaks” in E15 cortex chromatin and multi-tissue chromatin further supports the idea that a majority of these genes (19/27) are directly regulated by Pax6 in lens, although distinct biological materials were analyzed (E9.5 lens placodes for RNA, and newborn lenses for chromatin studies). For example, c-Maf, MafB, Pax6, Sfrp2, Tnc and Vcan/Cspg2 were previously identified as direct Pax6 targets in the lens [26], [36], [48], [53], [54]. Previously, we have shown down-regulation of c-Maf and Pax6 in the Pax6 null ectoderms [48]. Expression of Fat4, Trpm3, Pax6, Has2, Efnb2 and Nav1 was evaluated using qRT-PCR as shown in Figure 3B. Taken together, these results suggest five novel direct targets of Pax6 in lens.

We next asked if genes containing Pax6 co-occupied promoters show any special feature related to their expression levels in different tissues, and how many of them are regulated by Pax6. It has been proposed that functionally significant binding of transcription factor correlates with expression levels of surrounding genes [6]. To follow this point, we performed RNA expression profiling using the same tissues probed in the ChIP-chip studies (P1 lens, E15 cortex and β-cells). In addition, we included mouse embryonic stem (ES) cells, as a “reference” tissue for the origin of embryonic development, and Pax6-positive radial glia progenitors generated from mouse ES cells [55]. Three sets of biological replicates were used for RNA expression profiling of these five tissues/cells measured by microarray hybridization with Affymetrix Mouse Genome 430A 2.0 Arrays. Principal component analysis was performed and nearly 88% of the information was explained with three principal components (Figure S1A). The analysis showed that lens, forebrain and β-cell transcriptomes are distinct, with lens being closer to the forebrain than to β-cells. We next performed RNA expression analysis with data normalized to the expression levels in mouse ES cells. Analysis of transcripts linked to the 133 commonly occupied promoter regions identified above revealed that ∼90% of these genes undergo strong up- or down-regulation in all three tissues examined (Figure S1B). Concerning the question whether these genes are regulated by Pax6, we found 11 genes: *Cenpn*, *Gaa* and *Snca* (Figure 2 and Figure S1B), and *Adprhl2*, *Cap1*, *Cma2*, *Dpm2*, *Hook2*, *Rffl*, *Sra1* and *Trove2* (Figure S1B). In addition, *Otx2*, a gene regulated by Pax6 in the optic cup [47], is present in the list of 133 common genes, but was not identified through RNA expression profiling in lens. It is interesting to note that all 12 genes are highly regulated relative to their expression in ES cells, consistent with the view that Pax6 establishes novel cell lineages and controls cell differentiation [56], [57], [58]. From these data we conclude that, as expected, transcriptomes of these tissues/cells are globally different between lens, forebrain and β-cells. The genomic regions with Pax6 presence in all three types of chromatin are mostly linked to genes activated or repressed in differentiated cell types, compared to their expression levels in ES cells. Although the present data suggest that only 12 (9%) of these genes are Pax6-direct targets, they represent a pool of genes that could be regulated by Pax6 in many other developmental conditions in addition to those examined here (newborn lens and E9.5 lens placode).

### Kif1b and Snca are novel Pax6 direct target genes in lens and forebrain

**Figure 4:**
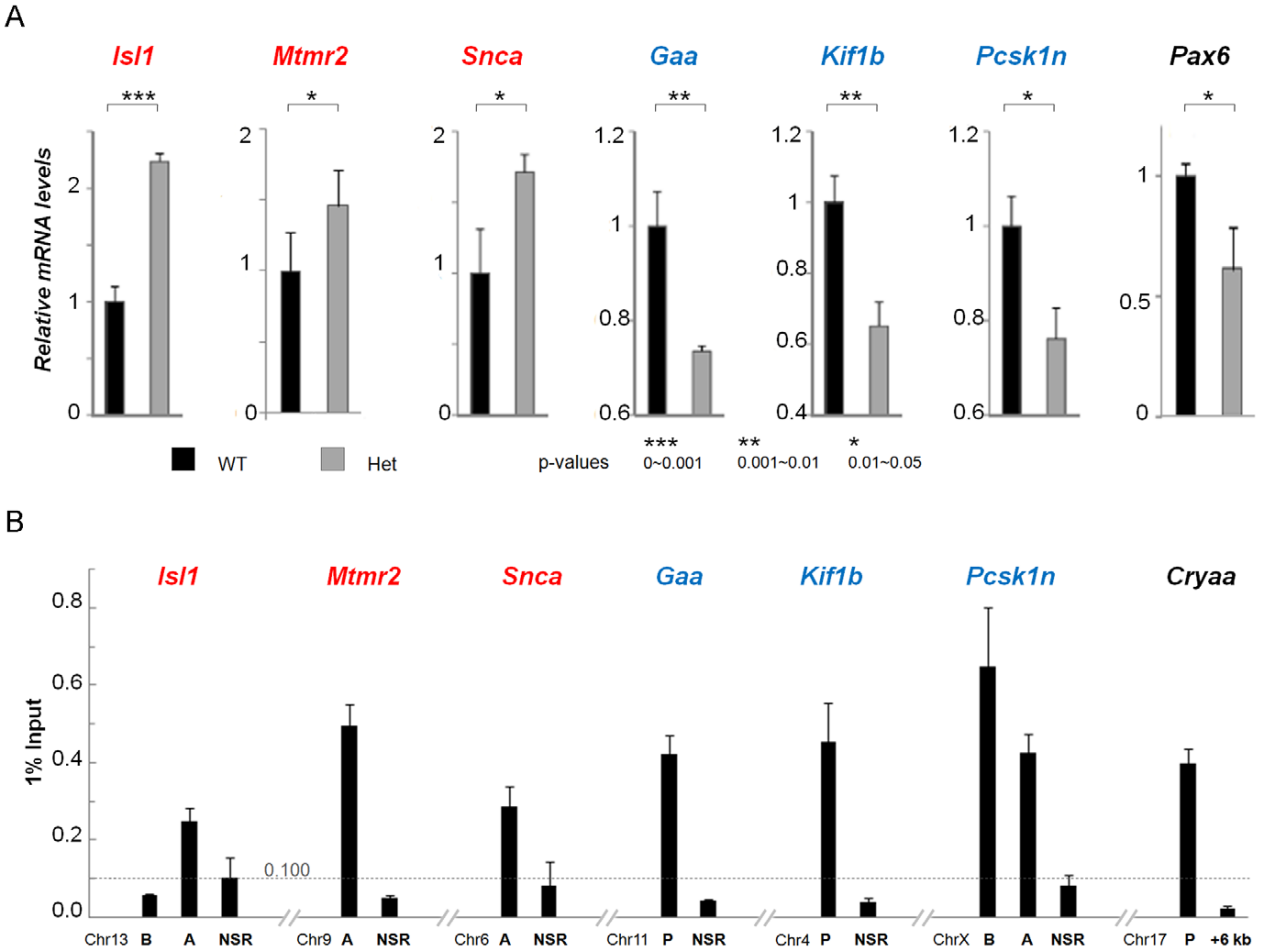
Verification of microarray results by qRT-PCR and qChIP. (A) Relative expression levels of *Gaa*, *Has2*, *Isl1*, *Kif1b*, *Mtmr2*, *Pax6*, *Pcsk1n*, *and Snca* in wild type (WT, shown in black) and Pax6^+/−^ (shown in gray) lenses were determined using qRT-PCR as described in Methods. B2m, Hprt and Ccni transcripts were tested as internal references, and all were found unchanged between the WT and Pax6^+/−^ lenses. The data are expressed relative to the unchanged expression level of B2m transcripts. For statistical evaluation of the results, p-values were calculated from paired Student t-tests. (B) Validation of Pax6-binding regions in lens chromatin by qChIPs. A and B are distal regions with Pax6 binding identified in ChIP-Chip experiments and P regions are binding regions around the proximal promoters. At each of these gene loci, a non-specific region (negative signals in ChIP-Chip experiments and no candidate Pax6 binding sites predicted) was also included as a negative control. In addition, Cryaa promoter (Cryaa-P) and +6 kb region serve as positive and negative controls respectively. The specific enrichments of Pax6 binding were detected at Isl1-A, Mtmr2-A, Snca-A, Gaa-P, Kif1b-P, Pcsk1n-B and Pcsk1n-A regions. The calculation of the cutoff value (0.100 of 1% input) for background signals and specific binding signals is described in Materials and Methods.

We next focused on the analysis of 76 genes (Figure 2) identified by RNA and ChIP-chip in newborn lens. *Gaa* (acid glucosidase α), *Kif1b* (kinesin family member 1B) and *Pcsk1n* (proprotein convertase subtilisin/kexin type 1 inhibitor) were activated by Pax6 in the lens, while *Isl1* (islet-1), *Mtmr2* (myotubularin related protein 2) and *Snca* (α-synuclein) were repressed by Pax6 in the lens. These genes were selected for further validation, as *Gaa*, *Kif1b*, *Isl1* and *Snca* are also regulated by Pax6 in embryonic cortex [44], [45], *Mtmr2* and *Pcsk1n* have strong expression in the mouse retina, and all regulatory elements for these genes have multiple putative Pax6-binding sites (summarized in Figures S2 and S3). In this group of six genes, *Gaa* and *Snca* showed Pax6 peaks in all three chromatin samples (Figures 1A and S3B). *Isl1* and *Kif1b* displayed Pax6-bound peaks in lens and β-cells, while *Mtmr2* and *Pcsk1n* showed peaks in lens chromatin only (Figures 1 and S2A). To validate the RNA expression data, we performed quantitative RT-PCR of these six genes (Figure 4A). Reduced Pax6 expression in Pax6^+/−^ lenses is shown as a control. The data confirmed that expression of these genes is dependent on Pax6. To validate Pax6 binding in promoter regions of these genes, quantitative ChIPs (qChIPs) were performed using primers corresponding to the “peak” regions (Figure S2) and surrounding non-specific regions (NSR) in the same locus. The mouse Cryaa gene promoter and the +6 kb regions were used as positive and negative controls respectively [39]. The results confirmed selective binding of Pax6 at all genomic regions identified by the ChIP-chip data except for the region ISL1-B at −5 kb of the *Isl1* locus (compare Figure 4B and Figure S2A). IgG control included in the experiments shows random background binding to these regions tested (Figure S4).

**Figure 5:**
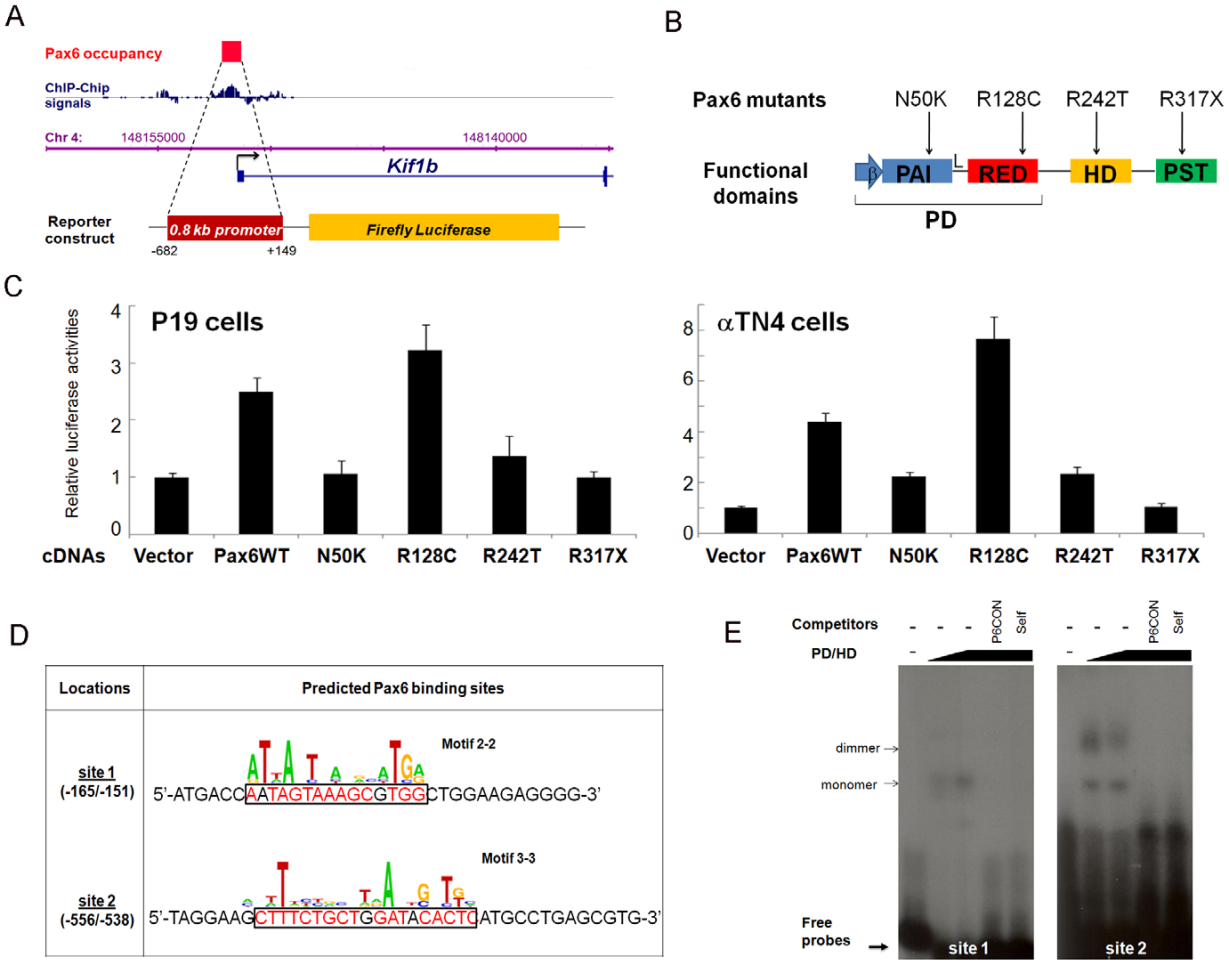
Pax6 regulates expression of Kif1b. (A) Identification of Pax6-binding regions by ChIP-Chip in lens chromatin and a corresponding luciferase reporter construct for transfection assays. (B) A diagrammatic summary of Pax6 mutants, N50K, R128C, R242T and R317X. β, N-terminal β-turn unit; PD, paird domain; PAI, N-termianl subdomian of PD; RED, C-terminal subdomian of PD; L, linker region; HD, homeodomain; PST, proline-serine-threonine rich transactivation domain. (C) Pax6 activates *Kif1b* promoter in cultured cells. Transient transfections were performed in P19 embryonic carcinoma and in αTN4-1 lens cell as described in Methods. (D) Prediction of Pax6 binding sites with novel Pax6 DNA binding motifs [48]. (E) EMSA validation of Pax6 binding to two probes identified by motifs 2-2 and 4-1. PD/HD, recombinant Pax6 protein containing both Pax6 paired domain (PD) and homeodomain (HD). P6CON, DNA-binding concensus for Pax6 paired domain.

**Figure 6:**
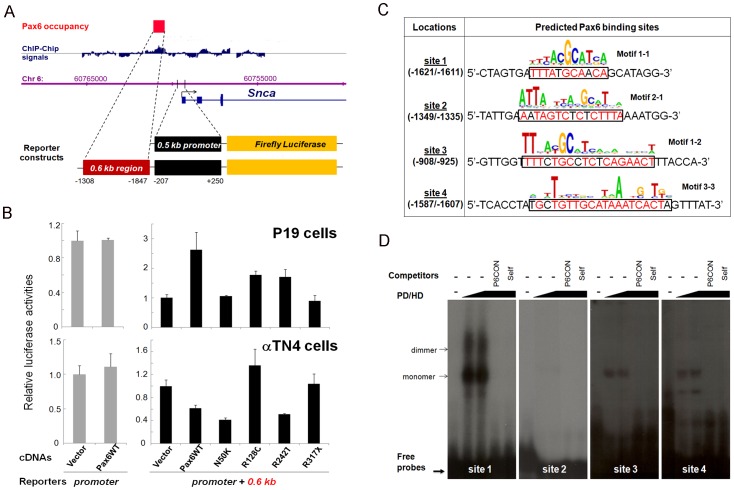
Pax6 regulates expression of Snca. (A) Identification of Pax6-binding region by ChIP-Chip in lens chromatin and corresponding luciferase reporter constructs for transfection assays. (B) Pax6 regulates *Snca* promoter/distal region in cultured cells. Transient transfections were performed in P19 embryonic carcinoma and in αTN4-1 lens cell as described in Methods. (C) Prediction of Pax6 binding sites with novel Pax6 DNA binding motifs [48]. (D) EMSA validation of Pax6 binding to the probes identified by motif 1-1, 1-2 and 3-3. PD/HD, recombinant Pax6 protein containing both Pax6 paired domain (PD) and homeodomain (HD). P6CON, DNA-binding concensus for Pax6 paired domain.

To further test whether Gaa, Isl1, Kif1b, Mtmr2, Pcsk1n, and Snca, are regulated by Pax6, we performed co-transfection studies in cultured P19 embryonic carcinoma and αTN4-1 lens epithelial cells. Eleven luciferase reporter constructs were co-transfected with Pax6. Pax6-binding regions, identified by ChIP-chip studies, pointed to multiple regulatory regions such as proximal promoters and distal regions, the putative enhancers. We found that *Kif1b* promoter and *Snca* promoter/enhancer were activated by Pax6 in P19 cells (Figures 5C and 6B). The *Kif1b* promoter was also activated in cultured lens epithelial cells (Figures 5C). In contrast, the *Snca* promoter/enhancer reporter plasmid was repressed by Pax6 in the cultured lens epithelial cells (Figure 6B). This finding is consistent with increased expression of *Snca* in Pax6 heterozygous lenses (Figure 4A). To test which parts of Pax6 molecule are involved in Pax6-mediated reporter gene activation and repression, we used a panel of four mutants, N50K, R128C, R242T and R317X, as shown in Figure 5B. Studies of Kif1b promoter show reduced activation by N50K and R242T mutants (Figure 5C) consistent with the presence of PAI and HD motifs in site 1 and 2, respectively (Figure 5D). Activation of Snca promoter/enhancer in P19 cells was reduced by all four mutants tested in agreement with complex nature of sites 1, 3 and 4 (Figure 6C). In contrast, transcriptional repression of this reporter gene construct required the presence of C-terminal domain (Figure 6B, data for R317X). The remaining four reporter genes (*Gaa*, *Isl1*, *Mtmr2* and *Pcsk1n*) were regulated by Pax6 within a range of 10–25% (data not shown). Next, we performed EMSAs with multiple binding sites identified in the *Kif1b* (Figures 5D and E) and *Snca* Pax6-binding regions, respectively (Figures 6C and D). The data showed binding of recombinant Pax6 proteins to two probes at the *Kif1b* locus and three probes at *Snca* locus. Use of specific oligonucleotide competitors confirmed the formation of specific Pax6-DNA complexes.

**Figure 7:**
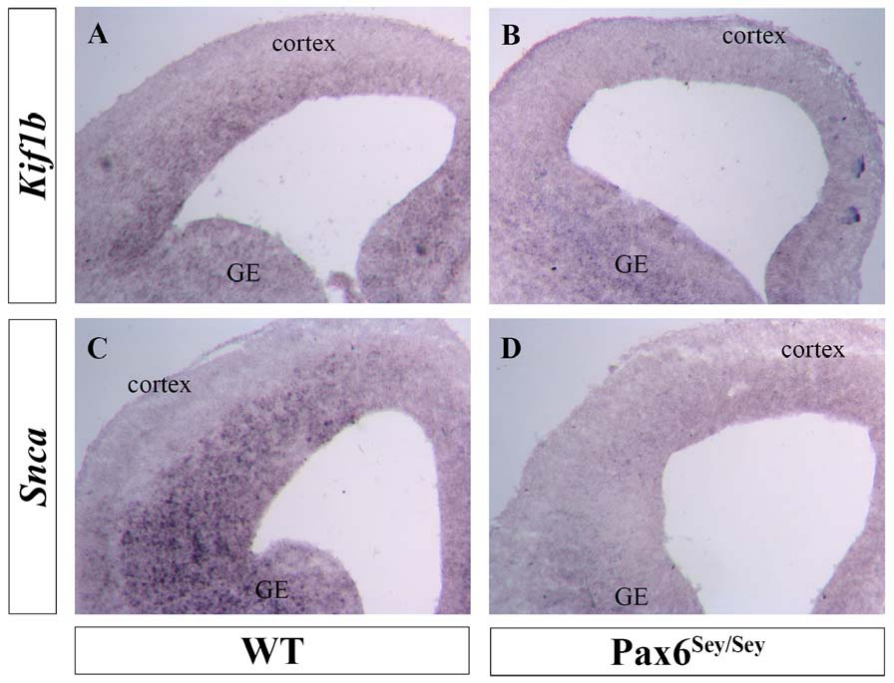
Down-regulation of Kif1b and Snca in Sye/Sey forebrains. In situ hybridization analysis of E14 wild type (WT) and Pax6^Sey/Sey^ mouse embryos. Ganglionic eminence, GE.

Finally, we performed *in situ* hybridization to evaluate the expression of Kif1b and Snca in the telencephalon of embryonic day 14 wild type (WT) and Pax6 null (*Sey/Sey*) mice. Notably, we detected both of these transcripts in the ventricular zone (VZ) of the WT cerebral cortex (Figures 7A and C), where Pax6 expression is highest, and this signal was virtually lost in *Sey/Sey* littermates (Figures 7B and D). Interestingly, Kif1b expression is not affected in the VZ of the neighboring lateral ganglionic eminence (Figures 7A and B), which expresses lower levels of Pax6 in WT. We suggest that *Kif1b* and *Snca* are directly regulated by Pax6 in lens and forebrain.

## Discussion

In this study, we focused on two questions central to understanding embryonic development: 1) Where are lineage-specific DNA-binding transcription factors, commonly expressed in different tissues, localized in chromatin from different tissues, and 2) which genes are directly regulated by the lineage-specific regulatory gene, Pax6, in the lens and forebrain? Answering these questions is important to understand the relationship between the binding of lineage-specific transcription factors in chromatin from different tissues and for the identification of developmental programs directly controlled by Pax6.

### Functional Pax6-binding in chromatin: Primary and secondary Pax6 target genes

Our data identified 133 promoter regions occupied by Pax6 in chromatin obtained from the lens, forebrain and pancreatic β-cells. We also found 868 genes that were occupied by Pax6 in two of these tissues. In summary, 1,001 genes (nearly 1/5 of genes recognized by Pax6) are co-regulated in these tissues. These data show that Pax6 binds to common genomic regions in different cellular contexts. These tissues represent distinct cell types, as further shown here by analysis of their transcriptomes. The combination of RNA expression profiling with ChIP-chip results yielded a relatively small number of candidate direct target genes, compared to the number of genes occupied by Pax6. The simplest interpretation is that RNA profiling identifies a mixture of primary (direct, 14% in newborn lens) and secondary (indirect) targets with the number of secondary targets exceeding the number of direct target genes. Nevertheless, there may be at least four reasons that explain the relatively low number of the directly regulated genes. First, the RNA analysis in Pax6 heterozygous lenses identified only those genes that are sensitive to Pax6 haploinsufficiency. A conditional gene approach to delete Pax6 at the lens vesicle/primary lens fiber cell formation (E12.5) could resolve this problem [25]. However, an ideal experiment to analyze appropriate chromatin from E12.5 primitive lens is technically challenging due to tissue microdissection and limited amount of chromatin obtained from small embryonic tissue. Second, the promoter ChIP-chip arrays restricted the analysis, because distal enhancers, which are major regulatory elements of tissue-specific and tissue-preferred genes, were not spotted on these arrays. This limitation is now fully addressable using ChIP-seq analysis [59]. Third, a significant fraction of the genome is occupied by specific transcription factors without any obvious regulatory function on known genes [6] as shown recently for retinoic acid nuclear receptors [60], MyoD and Myogenin [61], and the glucocorticoid receptor [62]. Finally, 10–20% of ChIP-chip and ChIP-seq data detect indirect interactions between the transcription factor and DNA [63].

On the other hand, the numbers of Pax6-bound promoters in lens, forebrain and β-cell chromatin appear relatively high compared to the total number of potentially transcriptionally active genes. It is possible that the current ChIP-chip data are partially compromised by a non-specific background signal. We could not fully address this question in the current study, however, many Pax6-binding regions were independently validated including six genes, Gaa, Isl1, Kif1b, Mtmr2, Pcsk1n, and Snca, shown here (Figure 4B), and five additional genes, Vcan/Cspg2, Mab21l2, Olfm3, Spag5 and Tgfb2 examined earlier [26]. Typically, genes selected for validation by qChIP are biased towards better understanding of Pax6-dependent gene regulatory networks. A recent ChIP-seq study using two sequential immunoprecipitations to identify binding of Pax3 and Pax7 in adult myoblasts revealed over 52,683 (4,648) loci enriched for Pax7 (Pax3), respectively [64]. Both Pax3 and Pax7 are structurally similar to Pax6. These findings suggest that Pax proteins, containing the internal homeodomain (HD), can recognize over 50,000 genomic loci due to the presence of five highly interactive DNA-binding modules (i.e. PAI, RED, β-turn, linker and HD) in the Pax3/4/6/7 protein subfamily that can recognize multiple different binding sites, as we have shown earlier for Pax6 and its splice variant Pax6(5a) [48]. In this regard, the number of Pax6-bound genes *in vivo* could actually be higher than that reported here, especially when one considers genes with Pax6-binding in enhancer regions.

### Genetic programs regulated by Pax6 in lens, forebrain and other tissues

Although a number of known Pax6-direct target genes provide molecular explanation of eye, forebrain and pancreas morphogenesis, the majority of genes controlled by Pax6 protein remain to be identified. In the lens, Pax6 appears to control lens development at multiple stages from the formation of lens lineage [52], establishment of the lens placode [20], control of the lens vesicle formation and its separation from the surface ectoderm [22], [23], regulation of cell cycle exit and differentiation of primary lens fiber cells [25] to the control of lens fiber cell denucleation via DNase IIβ [26], [65]. The present study adds six genes as novel Pax6-direct target genes *Gaa*, *Isl1*, *Kif1b*, *Mtmr2*, *Pcsk1n*, and *Snca* in the lens. A recent study has shown direct regulation of Pcsk1n by Pax6 in pancreas [66]. Combinations of RNA expression profiling with ChIP-chip studies and *in situ* hybridizations support the idea that Pax6 also regulates Gaa, Kif1b and Snca in the embryonic forebrain. Functions of Gaa, Kif1b and Snca have been established outside of the lens. Gaa is a lysozomal enzyme highly expressed in brain and its expression is increased in Pax6 mutant forebrain [45]. The kinesin motor protein Kif1b functions in the axonal transport of mitochondria and synaptic vesicles [67] and is required for outgrowth of specific long axons in the peripheral and central nervous systems [68]. Systematic studies of Kif1b as microtubule-based molecular motor systems in lens fiber cell elongation should open new research avenues onto this intricate process. Snca encodes α-synuclein, which regulates dopamine release and transport [69], [70], [71] and contributes to cognitive function [72]. Different regulation of Snca by Pax6 in lens and cortex is likely caused by different cell-specific contexts. The repressive function of Pax6 on Snca promoter/enhancer system in cultured lens cells requires the presence of Pax6 C-terminal domain. The repressor complexes bound to Pax6 through this subdomain remain to be identified.

Mutations in KIF1B and SNCA cause human diseases affecting the central nervous system. Human GAA mutations cause glycogen storage disease type II (Pompe's disease), manifestated by muscular dystrophy [73]. A single report of human aborted fetus identified large glycogen deposits within the eye excluding the iris epithelium and retinal pigmented epithelium [74]. Mutations in human KIF1B cause Charcot-Marie-Tooth disease type 2A [75] and have been linked to multiple sclerosis [76]. Finally, mutations in SNCA cause Parkinson's disease and Lewy body disease. Thus, it is possible that deregulation of GAA, KIF1B and SNCA is involved in pathology of human PAX6 patients with a range of neurological disorders including autism [77], [78], [79], cognitive disorders [80], epilepsy [81] and mental retardation [77].

**Figure 8:**
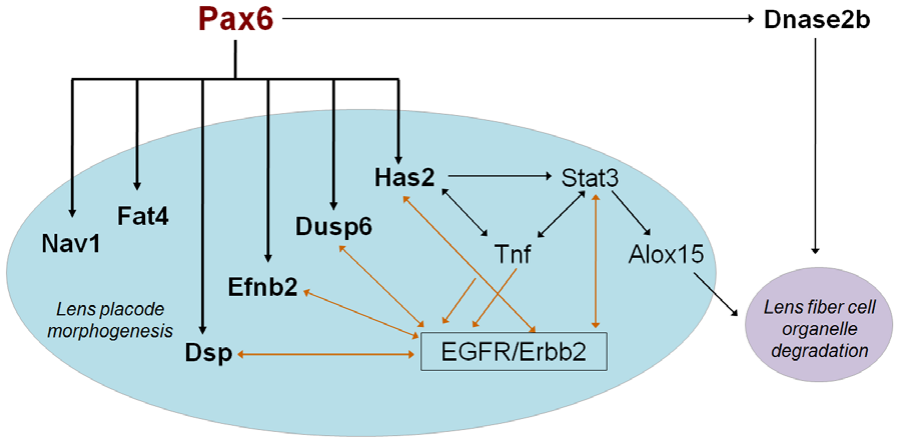
Diagrammatic summary of novel functions of Pax6 during lens development. The connections between genes regulated by Pax6 in lens placode were identified via Ingenuity Pathway Analysis (IPA) (Ingenuity Systems, Mountain View, CA). Expression of Stat3 is reduced in the Pax6 mutated E9.5 embryonic tissues. The Tnf promoter region is occupied by Pax6 in lens chromatin.

In the lens placode, the present data suggest Dsp, Dusp6, Efnb2, Fat4, Has2, Nav1 and Trpm3, as candidates for direct regulation by Pax6 with a range of potential functions in lens morphogenesis (Figure 8). Their Pax6-binding regions, identified by ChIP-chip studies, are shown in Figure S2. Dsp is an important component of cell-cell junction system in lens fibers [82], [83]. Dusp6 serves as a negative regulator of FGF/MAPK/Ras signaling [84], a key signal transduction pathway with multiple roles in lens lineage formation and lens fiber cell differentiation [85]. Ephrin-B2 (Efnb2), EPH-related receptor tyrosine kinase ligand 5, is specifically expressed in lens epithelium [86]. Similar proteins, Ephrin-A5 and EphA2, play a number of important roles in lens development and cataractogenesis [87]. Ephrins, including Efnb2, genetically interact with the Reelin pathway and control neuronal migration [88]. It has been shown in kidney, that Fat4 is a component of hippo signaling and important for planar cell polarity [89]; recent studies established roles of Wnt/planar cell polarity signaling in lens morphogenesis [90] and components of the Hippo pathway in regulating lens fiber cell terminal differentiation [91]. Hyaluronan synthase Has2 is a glycosyltransferase that catalyze polymerization of hyaluronan found in both intra- and extracellular compartments that protect epithelial cell integrity [92], [93]. Expression of Has2 was shown in the lens pit [94] and is an excellent candidate to better understand the molecular mechanisms of lens placode formation and its invagination [95], [96] as Has2 has been recently implicated in the otic placode morphogenesis as a FGF-regulated gene [97]. Nav1 is microtubule-associated protein involved in neuronal migration [98]. Given a number of similarities between genes regulated by Pax6 in lens and neuronal development [26], Nav1 may play similar roles in lens differentiation. Ingenuity Pathway Analysis performed on this gene set identified a hub comprised of three genes, Stat3 (down-regulated in the Pax6 mutated embryos), Tnf (occupied by Pax6 in lens chromatin) and EGFR/Erbb2 that link together downstream functions of Dsp, Dusp6, Efnb2 and Has2 (Figure 8). Future experiments to probe functions of these Pax6-regulated genes and precise identification of Pax6-binding sites within their regulatory regions will shed new light into the process of lens morphogenesis.

The RNA expression profiling analysis of wild type and Pax6 mutated embryos revealed several hundreds of novel transcripts whose expression is dependent on Pax6 during the formation of lens placode. Several examples (Figure 3A) deserve additional comments. Expression of Dnmt3a, an essential *de novo* DNA methylase [99], is not only reduced in Pax6 mutant surface ectoderm, but also in Pax6-deficient optic vesicles/forebrains isolated from E9.5 embryos [100]. Dnmt3a is expressed in E11.5 lens [101]. In contrast, expression of γB-crystallin (Crygb) and Nav1 is reduced in Pax6-deficient surface ectoderm but increased in mutant optic vesicles [100]. Both Dkk3 and Sfrp2 function as negative regulators of Wnt/β-catenin signaling [102]. Expression of Dkk3 is reduced in E12 Pax6 mutated cortex [45] and directly regulated by Pax6 [103]. The regulation of three canonical Wnt signaling modulators, Dkk1, Sfrp1 and Sfrp2, through Pax6 has already been shown in lens and during neurogenesis [54]. Expression of Cdh7 and Wnt5a is also Pax6-dependent in rat embryonic hindbrain [104]. In pancreas, both c-Maf and MafB (Figure 3A) were shown as Pax6-direct target genes [36]. In conclusion, considering the multitude of roles played by Pax6 in embryonic development through the control of cell lineage formation, cellular differentiation, neuronal and ocular stem cell biology [105], [106], [107] and tissue regeneration [108], future studies will require integration of the ChIP-seq and RNA expression profiling using smaller amounts of chromatin to elucidate function of this regulatory protein at different stages of embryonic development and at different stages. This will allow expanded reconstruction of gene regulatory networks (GRNs) under the genetic control of Pax6, including identification of unique pathways and those shared between two or more distinct cell types.

## Conclusions

Our results demonstrate that Pax6 interacts preferentially with ∼8.5 kb promoter regions examined through high density oligonucleotide arrays in a tissue-specific fashion though ∼20% of the regions identified are accessible to Pax6 in at least two tissues examined, i.e. lens, embryonic cortex and cultured pancreatic β-cells. Two combinations of ChIP-chip and RNA expression profiling in Pax6 mutated tissues suggest eleven novel direct targets of Pax6 in the lens. Six of these genes: *Gaa*, *Isl1*, *Kif1b*, *Mtmr2*, *Pcsk1n*, and *Snca*, were confirmed by studies in the newborn lens chromatin. Additional data indicate that Pax6 also regulates Kif1b and Snca in the embryonic forebrain. Collectively, these results provide new insights into the process of embryonic lens formation, reveal common Pax6-dependent pathways during lens and neuronal differentiation, and open new research avenues to dissect the Pax6-regulated processes at the molecular level.

## Materials and Methods

### Ethics Statement

Animal husbandry and experiments were performed following the approved protocol of the Albert Einstein College of Medicine Animal Institute Committee and the Association for Research in Vision and Ophthalmology (ARVO) for the use of experimental animals in ophthalmic and vision research.

### Cells and tissues

Lenses were isolated from newborn mice (strain CD-1), forebrains were obtained from E15.5 embryos, mouse βT3 pancreas cells were cultured as described elsewhere [109], Pax6-positive cortical progenitor cells (CA-5) were differentiated from mouse ES cells as described elsewhere [55]. The materials were stored in RNA Later (Ambion, Woodlands, TX).

### Oligonucleotide microarrays and mRNA expression profiling

RNA isolations were performed using the RNeasy MiniElute Kit and RNase-Free DNase set (QIAGEN, Valencia, CA). RNA quality was assessed using the Agilent 2100 Bioanalyzer with the Nano LabChip Kit (Agilent Technologies; Palo Alto, CA). Replicate sets of RNAs from mouse ES cells, Pax6-positive cortical progenitor cells, E15.5 forebrain, newborn lens and cultured βT3-cells (n = 3) were prepared for microarray analyses. cDNA synthesis and amplifications were performed with the Ovation ™ RNA Amplification System V2 (Nugen, San Carlos, CA) using 50 ng of total RNA per sample. Amplified cDNAs were cleaned and purified with the DNA clean and Concentrator ™-25 kit (Zymo Research, Orange, CA). Fragmentation and labeling was performed using the FL Ovation ™ cDNA Biotin Module V2 (Nugen, San Carlos, CA) according to manufacturer's instructions. The samples were subsequently hybridized on Mouse Genome 430A 2.0 Arrays (Affymetrix, Santa Clara, CA) following the manufacturer's specification.

Laser microdissections were conducted on sections prepared from E9.5 wild type and Pax6 null embryos followed by cDNA analysis via Illumina Mouse WG-6 V1 1 R4 expression BeadChips as described elsewhere [96]. Three sets of biological replicate experiments (E9.5), each comprised from independent triplicates, were entered into the initial analysis of the differentially expressed genes. In addition, we used additional triplicate experiment generated using E10.5 mouse embryos. The raw intensities were generated by the array scanner; the fold change (FC) calculations were based on median centered, baseline (WT) normalized data where the negative values (detected upon scanning as lower than the array background) were trimmed to the minimum intensity unit equal to 1. The KO FC calculations were expressed as the median FC value in the KO samples divided by the median FC in the WT samples as shown in Fig. 3A. Illumina GenomeStudio Diff Score thresholds (i.e. default program recommended by Illumina to analyze differential abundance of these arrays) were applied to this dataset as well to additional two independent triplicate datasets. This procedure includes log transformation and generates a Diff Score, a measure of transformation of the p-value that provides directionality to the p-value based on the difference between the average signal in the reference group vs. the comparison group. The numbers were generated at DiffScore ±13 corresponding to p-value<0.05. Expression of 27 individual genes was evaluated using all four biological triplicates (12 mutants vs. 12 wild types) by Illumina GenomeStudio Diff Score thresholds.

### ChIP-chip and qChIP assays

Chromatin was prepared from E15.5 forebrain, newborn lens and cultured βT3-cells, crosslinked and processed as described earlier [26]. Three sets of biological replicates were performed using each individual tissue. For the analysis, we used GeneChip Mouse Promoter 1.0R Arrays (Affymetrix) with over 28,000 mouse promoters. The data were analyzed using Model-based Analysis of Tiling-arrays [46], with the thresholds of MAT score of 4.0, fold-enrichment of 2, and 5% false discovery rate. The “Peaks” from MAT analysis were linked to individual genes using Cis-regulatory Element Annotation System (CEAS) [110]. Validation of data via qChIP was performed using lens chromatin as described elsewhere [48]. Pax6-binding regions were evaluated in three independent lens chromatin preparations. Briefly, the primers are designed for the ChIP-Chip peak regions and surounding non-specific regions (NSRs). The amounts of each specific DNA fragment in immunoprecipitates were determined by quantitative PCR reactions using a standard curve generated for each primer set with 0.04, 0.2, and 1% input DNA samples. The enrichments obtained with IgG were subtracted from the corresponding values obtained with anti-Pax6 antibody (Millipore). To determine a critical value to distinguish real specific binding signals from background noise, analysis of variance (ANOVA) was first performed for the signals obtained from all six NSRs and Cryaa +6 kb (negative controls), indicating no significant differences among these NSRs. We then defined these sites as the background group and performed a student t-test for the background group. The results showed the 99% confidence interval (CI) of the background signal value is 0.019∼0.100, indicating that all regions having signals higher than 0.100 should be considered as specific Pax6-binding regions. The primer sequences are listed in Table S2.

### Bioinformatic tools

The GO and KEGG pathway functional annotations were performed using the NIH web based tool DAVID (the Database for Annotation, Visualization and Integrated Discovery) (http://david.abcc.ncifcrf.gov/) [111]. Analysis of Pax6 DNA-binding motifs was performed using FIMO (Find Individual Motif Occurrences), a part of the MEME Suite software toolkit (http://meme.sdsc.edu/meme/cgi-bin/fimo.cgi) [51].

### Reporter plasmids and transient cotranfections

Wild type Pax6 expression clones in the pKW10 vector were described earlier [112]. Three Pax6 missense (N50K, R128C, R242T) and one nonsense (R317X) mutants are based on naturally occurring mouse [113] and human [114], [115], [116] mutations. The reporter plasmids contained homologous promoters cloned in pGL3 (Promega). For studies of Gaa, Isl1, Kif1b, Mtmr2, Pcsk1n and Snca, at least one distal region was cloned either 5′ or 3′ of the promoter depending on its natural localization in genome as shown in Figures 5, 6 and S2. The individual regions were synthesized by GenScript or amplified by PCR. Transient transfections were conducted by Lipofectamine 2000 (Invitrogen) in P19 embryonic carcinoma cells that do not express endogenous Pax6 proteins [117] and in αTN4-1 lens epithelium cells that express endogenous Pax6 proteins [118]. The cells were co-transfected with 0.6 µg of firefly luciferase reporter plasmids, 50 ng of the expression plasmid encoding Pax6 and corresponding empty vector pKW10, and 20 ng of Renilla-TK internal control using 24-well microplates. Dual-Luciferase Reporter Assay System (Promega) was used according to the manufacturer's instructions.

### RNA analysis: qRT-PCR and in situ hybridization

Relative expression levels of seven genes (*Gaa*, *Isl1*, *Kif1b*, *Mtmr2*, *Pax6*, *Pcsk1n*, and *Snca*), identified in Pax6 heterozygous lenses, and, six genes (*Fat4*, *Trpm3*, *Pax6*, *Has2*, *Efnb2*, and *Nav1*), identified in Pax6 null ectoderms, were verified by qRT-PCR. Total RNA from newborn wild type and Pax6^+/−^ littermates lens were isolated using RNeasy MiniElute Kit and RNase-Free DNase set (QIAGEN, Valencia, CA). Subsequently, cDNA was synthesized with Random Hexamer primers (Invitrogen) and Superscript TM III Reverse Transcriptase (Invitrogen), following manufacturer's instruction. cDNA was diluted 10 times and qRT-PCR was performed with Applied Biosystems (ABI, Foster City, CA) 7900HT fast Real-Time PCR system with Power SYBR Green PCR master mix (ABI). For E9.5 embryos, the total RNA was extracted from laser-capture dissected lens placodes. cDNA was synthesized and amplified by Ovation® Pico WTA Systems V2 (NUGEN). The quantitative Real-Time PCR was performed on Illunima ECO system with SYBR green for Nav1 and with Taqman fluorescent probe method for other genes. Transcripts encoding B2m (for new born lens) and Actb (for lens placode) genes were used for normalization of expression levels of tested genes in both wild type and mutant tissues as described earlier [26], [119]. Primers were designed using Primer 3 (http://frodo.wi.mit.edu/cgi-bin/primer3) and the sequences are listed in Table S2. In situ hybridization (ISH) analysis of Kif1b and Snca employed probes generated using the primers summarized in Table S2. ISHs were performed as previously described [120].

## Supporting Information

Figure S1 RNA expression profiling of 133 genes commonly occupied by Pax6 in three distinct chromatin sources. A) Principal component analysis of five tissues/cells. Total RNA samples were prepared from mouse embryonic stem cells (ESC, brown circles), radial glia-progenitor cells differentiated from ESCs (CA-5, red circles) [55], E15 embryonic cortex (blue circles), P1 lens (grey circles), and β-cells (βT3-cell line, green circles) [109], and subjected to analysis using the Mouse Genome 430 2.0 Arrays (Affymetrix, Santa Clara, CA) as three biological replicates. B) RNA expression profiling of 133 genes (see Figure 1A) in mouse ES, Pax6-positive radial glia progenitors (CA-5), E15 forebrain/cortex, newborn lens and β-cells. Hierarchical clustering of the expression data was performed using GeneSpring 7.2 (Agilent Technologies, Santa Clara, CA). Eight (*Adprhl2*, *Cap1*, *Cma2*, *Dpm2*, *Hook2*, *Rffl*, *Sra1* and *Trove2* – regulated by Pax6 in E9.5 lens placodes), three (*Cenpn*, *Gaa* and *Snca* – regulated by Pax6 in newborn lens) and *Otx2* (regulated by Pax6 in the optic cup) genes are labeled by blue, green and black squares, respectively.
(TIF)

Figure S2 ChIP-chip results of Isl1, Mtmr2, Snca, Kif1b, Pcskn1, Gaa and Dsp, Dusp6, Efnb2, Fat4, Has2, Nav1, Trpm3 in the chromatins of P1 lens, E15 cortex and β-cells.
(PDF)

Figure S3 A summary of Pax6-binding site motifs identified in 13 genes by both FIMO and mismatch searching. (A) Six target genes indetified in Pax6^+/−^ lens (Gaa, Isl1, Kif1b, Mtmr2, Pcsk1n, and Snca); and seven target genes in the lens placode (Dsp, Dusp6, Efnb2, Fat4, Has2, Nav1 and Trpm3). The red highlighted sequences were tested and validated in EMSA assay. (B) The consensus sequences and stardards used for mismatch searching. The mismatch searching was performed by online software Fuzznuc (http://mobyle.pasteur.fr/).
(PDF)

Figure S4 IgG randomly binds to the genomic loci in qChIP experiments. IgG (the same amount as Pax6 antibody) was used for IP as a control in each of the three biological repeats of qChIP experiments. While the specific enrichments of Pax6 binding at particular genomic regions were repeatedly detected (Figure 4), the non-specific binding by IgG are randomly distributed at the tested genomic regions between the independent experiments.
(TIF)

Table S1 A compilation of genes occupied by Pax6 in chromatin prepared from β-cells, forebrain and lens.
(XLS)

Table S2 Summary of oligonucleotides used in this study.
(PDF)
