# Supplementary figures and images for: Pax6 Interactions with Chromatin and Identification of Its Novel Direct Target Genes in Lens and Forebrain
Source: PLoS One. 2013 Jan 14;8(1):e54507. doi: 10.1371/journal.pone.0054507 (PMC3544819; doi:10.1371/journal.pone.0054507)

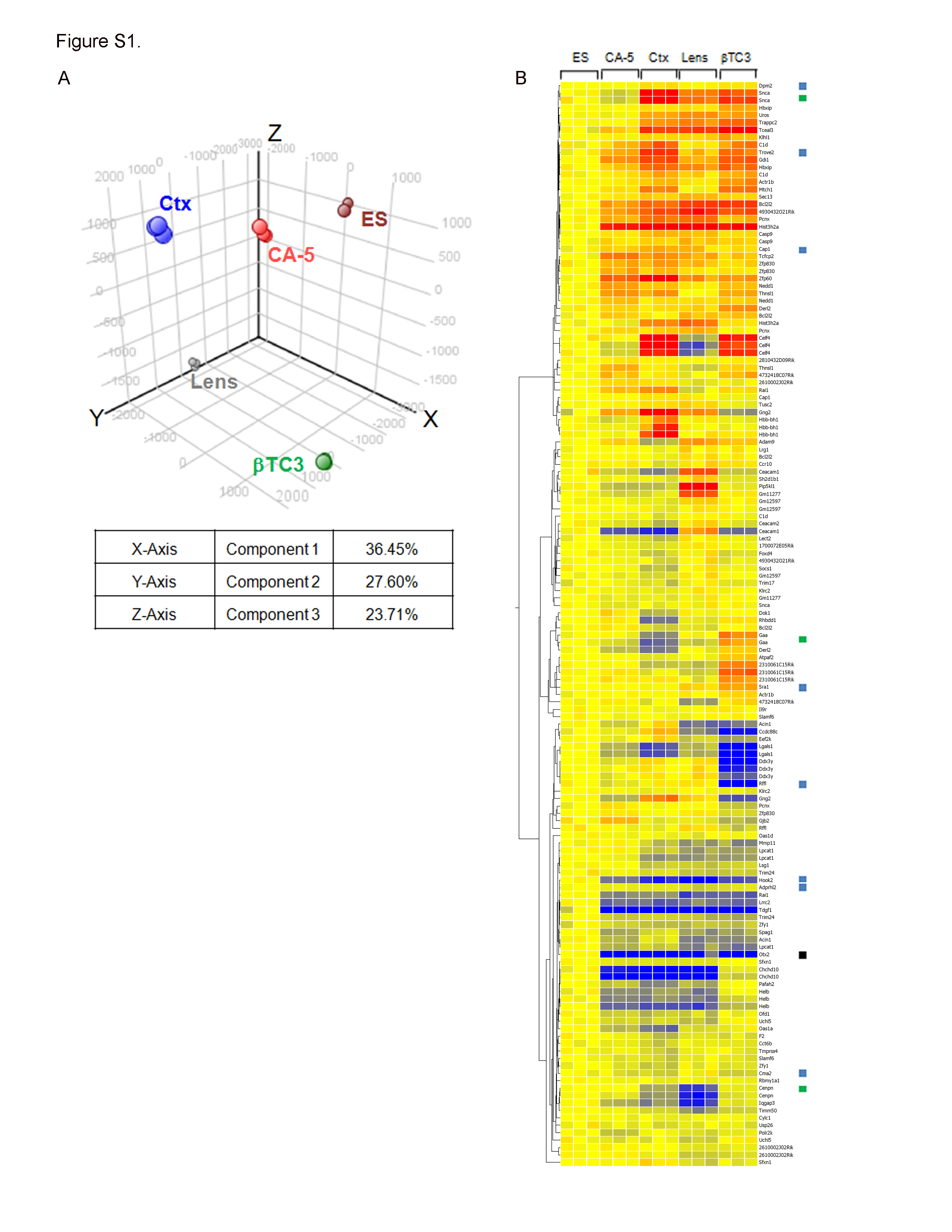

Supplement: Figure S1 — RNA expression profiling of 133 genes commonly occupied by Pax6 in three distinct chromatin sources. A) Principal component analysis of five tissues/cells. Total RNA samples were prepared from mouse embryonic stem cells (ESC, brown circles), radial glia-progenitor cells differentiated from ESCs (CA-5, red circles) [55], E15 embryonic cortex (blue circles), P1 lens (grey circles), and β-cells (βT3-cell line, green circles) [109], and subjected to analysis using the Mouse Genome 430 2.0 Arrays (Affymetrix, Santa Clara, CA) as three biological replicates. B) RNA expression profiling of 133 genes (see Figure 1A) in mouse ES, Pax6-positive radial glia progenitors (CA-5), E15 forebrain/cortex, newborn lens and β-cells. Hierarchical clustering of the expression data was performed using GeneSpring 7.2 (Agilent Technologies, Santa Clara, CA). Eight (Adprhl2, Cap1, Cma2, Dpm2, Hook2, Rffl, Sra1 and Trove2 – regulated by Pax6 in E9.5 lens placodes), three (Cenpn, Gaa and Snca – regulated by Pax6 in newborn lens) and Otx2 (regulated by Pax6 in the optic cup) genes are labeled by blue, green and black squares, respectively. (TIF) [file pone.0054507.s001.tif]

Figure S2

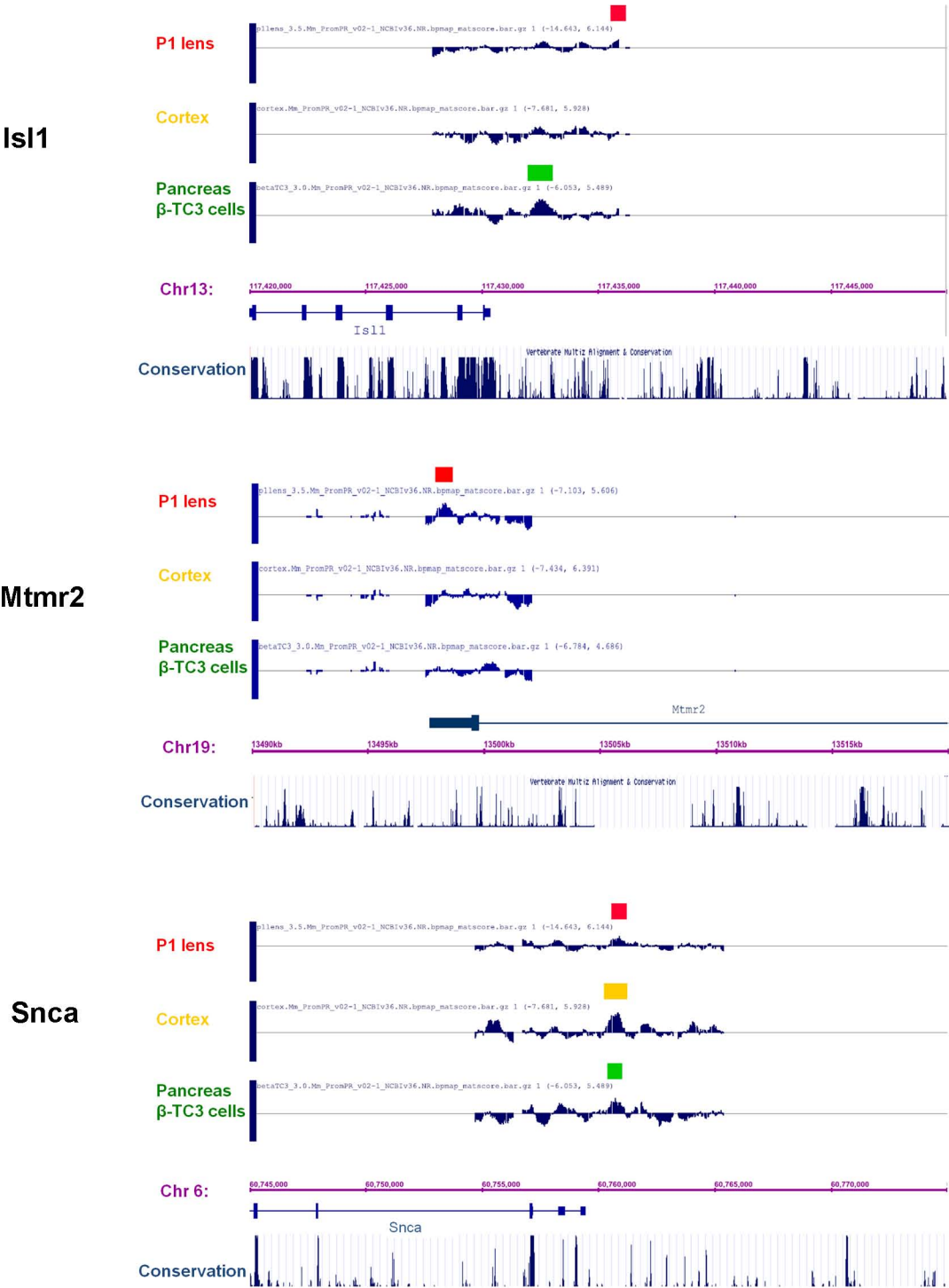

Figure S2

Kif1b

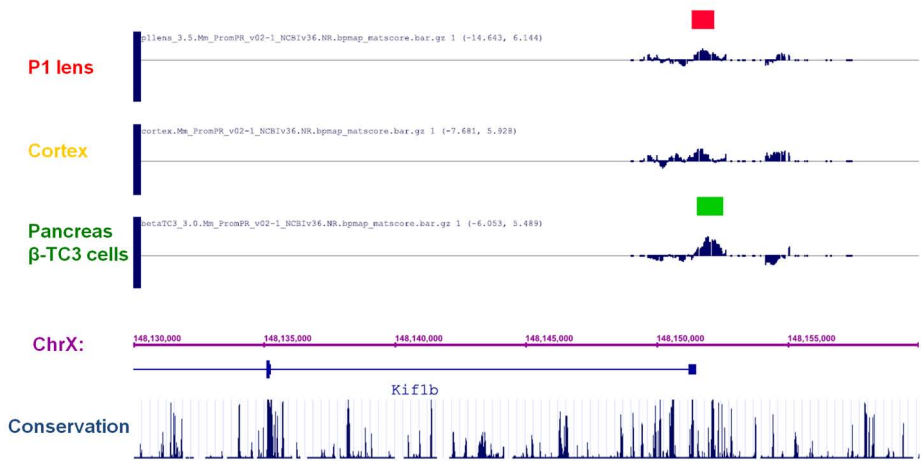

Pcskn1

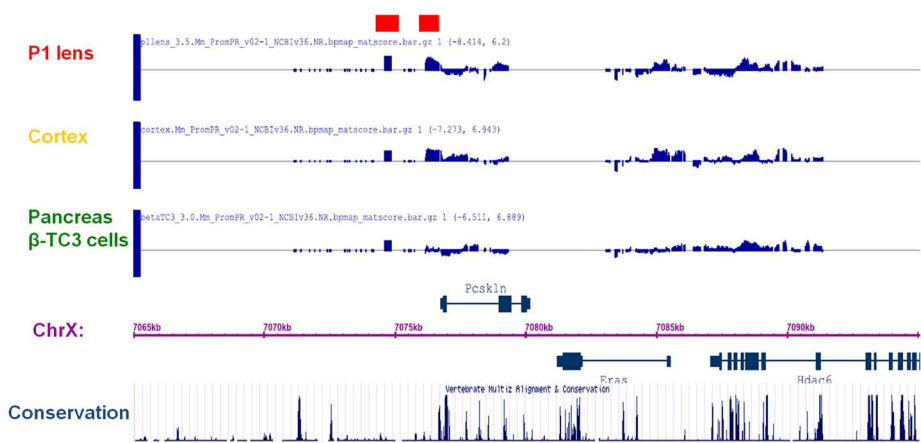

Gaa

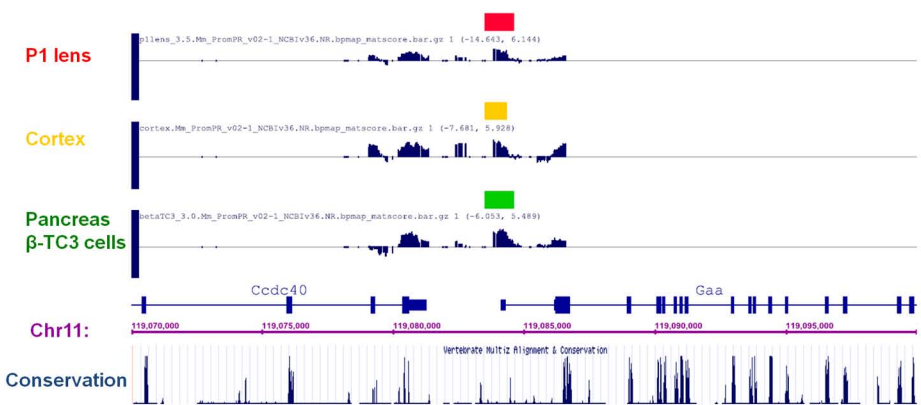

Figure S2

Dsp

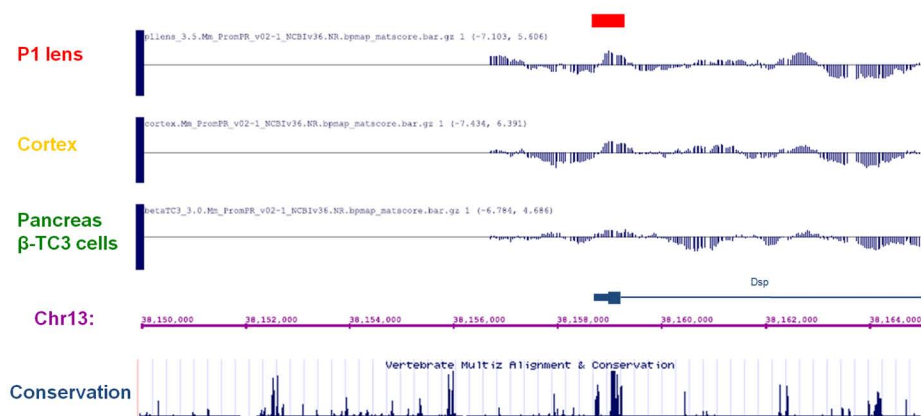

Dusp6

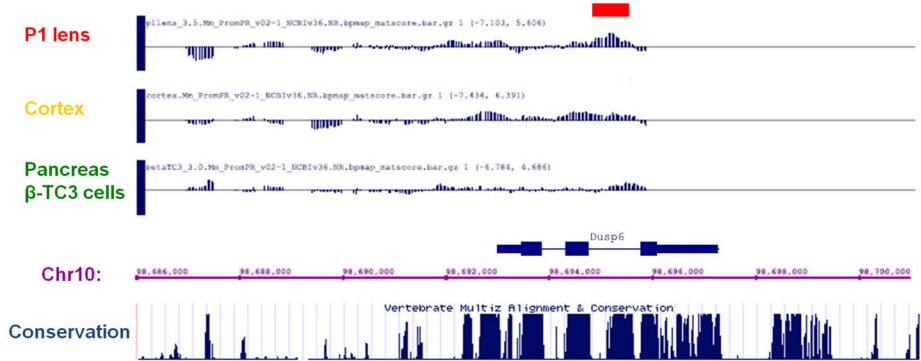

Efnb2

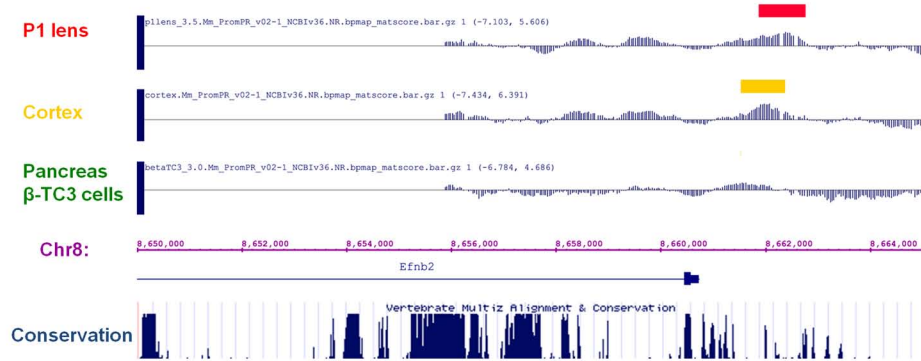

Fat4

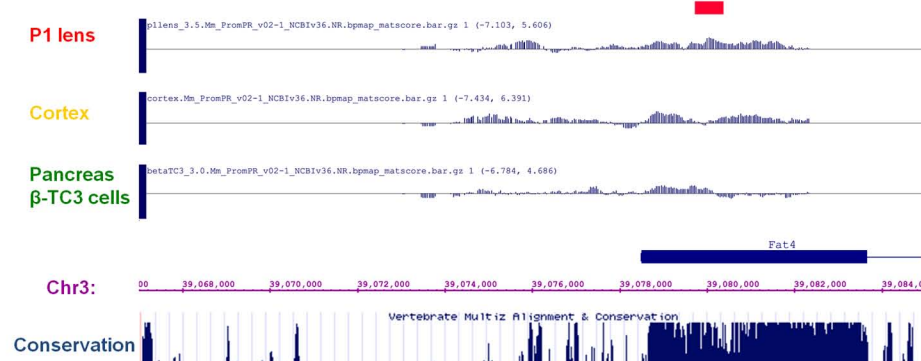

Figure S2

Has2

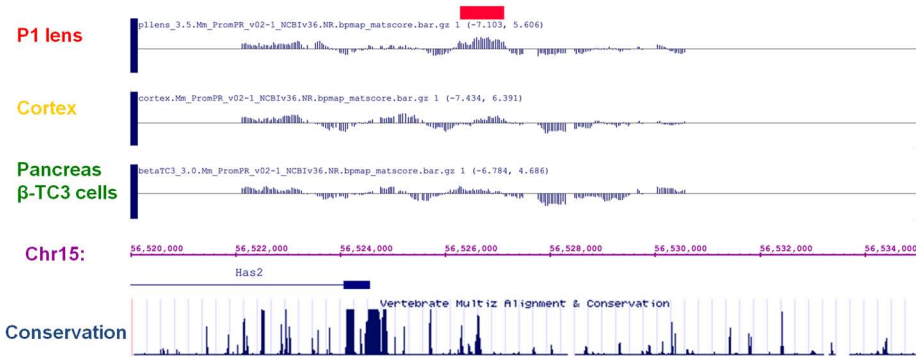

Nav1

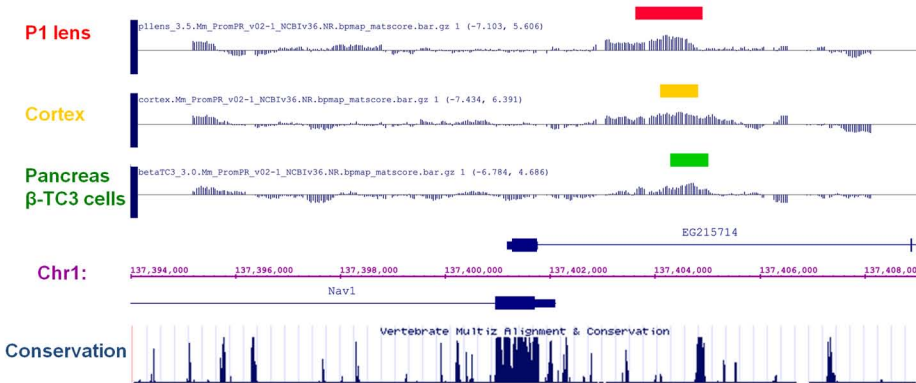

Trpm3

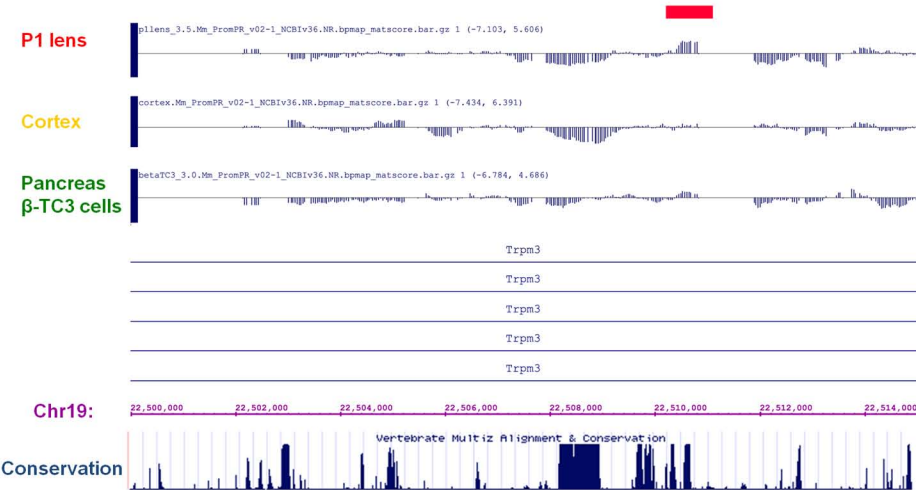

Supplement: Figure S2 — ChIP-chip results of Isl1, Mtmr2, Snca, Kif1b, Pcskn1, Gaa and Dsp, Dusp6, Efnb2, Fat4, Has2, Nav1, Trpm3 in the chromatins of P1 lens, E15 cortex and β-cells. (PDF) [file pone.0054507.s002.pdf]

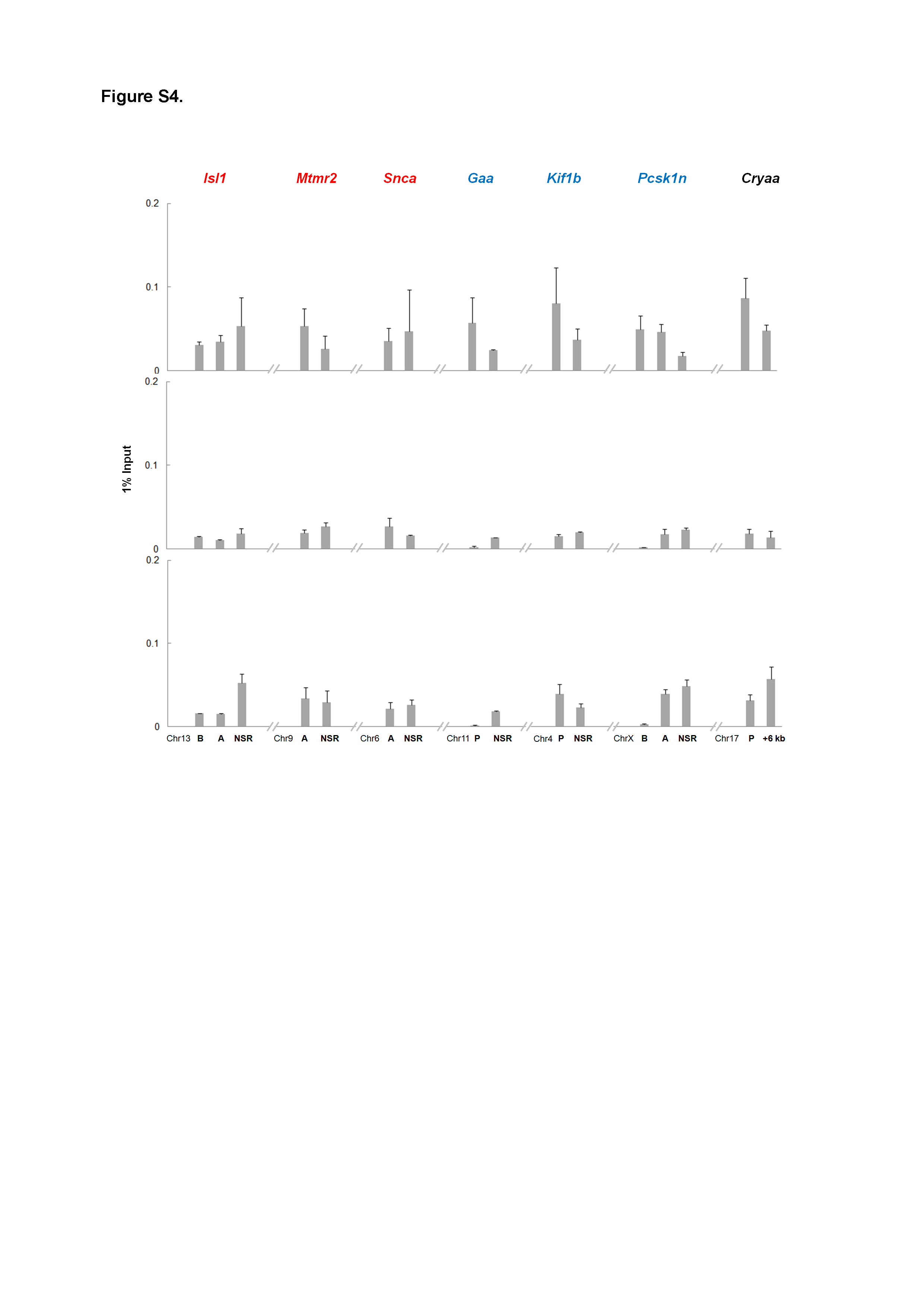

Supplement: Figure S4 — IgG randomly binds to the genomic loci in qChIP experiments. IgG (the same amount as Pax6 antibody) was used for IP as a control in each of the three biological repeats of qChIP experiments. While the specific enrichments of Pax6 binding at particular genomic regions were repeatedly detected (Figure 4), the non-specific binding by IgG are randomly distributed at the tested genomic regions between the independent experiments. (TIF) [file pone.0054507.s004.tif]
