# Supplementary material for: Pax6 Interactions with Chromatin and Identification of Its Novel Direct Target Genes in Lens and Forebrain
Source: PLoS One. 2013 Jan 14;8(1):e54507. doi: 10.1371/journal.pone.0054507 (PMC3544819; doi:10.1371/journal.pone.0054507)
Supplement: Figure S3 — A summary of Pax6-binding site motifs identified in 13 genes by both FIMO and mismatch searching. (A) Six target genes indetified in Pax6+/− lens (Gaa, Isl1, Kif1b, Mtmr2, Pcsk1n, and Snca); and seven target genes in the lens placode (Dsp, Dusp6, Efnb2, Fat4, Has2, Nav1 and Trpm3). The red highlighted sequences were tested and validated in EMSA assay. (B) The consensus sequences and stardards used for mismatch searching. The mismatch searching was performed by online software Fuzznuc (http://mobyle.pasteur.fr/). (PDF) [file pone.0054507.s003.pdf]

Figure S3

A

Loci	Sites	Sequences	Motifs	Methods
**Isl1 (A)**	(1)	TTCATTTACATTTCAAAT	1-3	Fuzzuc
	(2)	GCCTAATTGGCCA	3-1	Fuzzuc & FIMO
	(3)	TCACCCCTTTATTAAC	3-2	FIMO
**Isl1 (B)**	(4)	AAATTGTCCCAATGATCT	1-3	Fuzzuc
	(5)	TTAACACATCAAGCGTAT	3-2	FIMO
**Mtmr2**	(1)	TCATTTCACTATTTATTT	1-3	Fuzznuc & FIMO
	(2)	TTAATTTAAAACTTGGTT	1-3	Fuzznuc
	(3)	ATAACTTGTGCATGA	2-1	Fuzznuc
	(4)	AAATAATTGCTTA	3-1	Fuzznuc & FIMO
	(5)	AAATCTTTCATTGG	4-1	Fuzznuc
	(6)	TTCACGATAGAATG	P6CON	Fuzznuc
**Snca**	(1)	TTTATGCAACA	1-1	Fuzznuc
	(2)	AATAGTCTCTCTTTA	2-1	Fuzznuc
	(3)	TTATTTCTCTTTTA	4-1	Fuzznuc
	(4)	TTTCTGCCTCTCAGAACT	1-2	FIMO
	(5)	TGCTGTTGCATAAATCACTA	3-3	FIMO
**Kif1b**	(1)	AATAGTAAAGCGTGGCTGGAA	2-2	Fuzznuc
	(2)	GCTTTCTGCTGGATACACTC	3-3	Fuzznuc
	(3)	TCATTGTCCGTTTA	4-1	Fuzznuc & FIMO
	(4)	CTCAGGCATGAGTG	P6CON	Fuzznuc
**Pcsk1n (A)**	(1)	TTTAATTTCACTTTA	2-1	Fuzznuc
	(2)	TATTTGTTTGTTTA	4-1	Fuzznuc
	(3)	TTATTATTTATTTT	4-1	FIMO
**Pcsk1n (P)**	(4)	TTCTGGCATGA	1-1	Fuzznuc & FIMO
	(5)	TTTATCCATCA	1-1	Fuzznuc
	(6)	AACATTTACATATTTGTT	1-3	Fuzznuc
	(7)	TAAGCTCCTACTTAAT	3-2	Fuzznuc
	(8)	ATTGTTTATCCATCA	2-1	FIMO
	(9)	ATAAACAATTACTGGCTGCAT	2-2	FIMO
	(10)	AATTGCCACAGAATACCTGT	3-2	FIMO
	(11)	CAGTAATTGTTTA	3-1	FIMO
	(12)	ATCATTTAAAAAACATTT	1-3	FIMO
**Gaa**	(1)	CTCAAGCTTGCTTT	P6CON	Fuzznuc
**Dusp6**	(1)	TTTAAGCAAGA	Motif1-1	Fuzznuc
	(2)	ATAAGGAAAACATGGCTGATT	Motif2-2	Fuzznuc & FIMO
	(3)	AAATGGCTCAAATA	Motif4-1	Fuzznuc
	(4)	TATTTTTTTAATTA	Motif4-1	Fuzznuc
	(5)	TTCTTGCTTAAAGGGAAA	M1-2	FIMO
**Efn2**	(1)	TTCACGCTAGGTTA	P6CON	Fuzznuc
	(2)	AGTTTTTCCAAACCAATT	1-3	FIMO
**Has2**	(1)	TTCAAGCAGCA	1-1	Fuzznuc
	(2)	TTTCATCACACTTAA	2-1	Fuzznuc
	(3)	GATTAATTGTTTT	3-1	Fuzznuc
	(4)	AAATTGCTATTTTA	4-1	Fuzznuc
	(5)	TAATTGTTTTTGTA	4-1	Fuzznuc
**Nav1**	(1)	TTCACTCATGC	1-1	Fuzznuc
	(2)	TTTAGGAATCA	1-1	Fuzznuc
	(3)	AATATTTCCCTGACTCCATC	3-3	Fuzznuc
	(4)	CACAAGCATGAGTG	P6CON	Fuzznuc
**Trmp3**	(1)	TCCTTTTACCGTTGATTT	1-3	Fuzznuc
	(2)	GCTTAGGTAATTC	3-1	Fuzznuc
	(3)	TGCTGTCTTAGTTACT	3-2	Fuzznuc
	(4)	AAATTCCTTATTTA	4-1	Fuzznuc
	(5)	TTAATGTTCAAATG	4-1	Fuzznuc
	(6)	AACAAGCATGACTT	P6CON	Fuzznuc
	(7)	TCCTCCCATGACTT	P6CON	Fuzznuc
	(8)	TTTACCGTTGATTT	P6CON	FIMO

Figure S3

B

Motifs	Sequences	Length (nt)	Conserved nucleotides	Mismatch allowed
1-1	TTYACGCATSA	11	10	2
1-2	TTNAYGCATCANANCTCW	18	14	3
1-3	WNMWTTYMCNNMTNRNNT	18	8.5	1
2-1	ATTANTYACGCWTNA	15	12	3
2-2	ATTANTNANGCATGRNNRNWY	21	13	3
3-1	GCNTAATTRNTTM	13	10	2
3-2	TNNNSNCNTAATTNNT	16	8.5	1
3-3	MNTTTYYGCNTRANTSNYKC	20	12.5	3
4-1	TAATTGYTCAWTKR	14	12	3
