# Supplementary material for: Pax6 Interactions with Chromatin and Identification of Its Novel Direct Target Genes in Lens and Forebrain
Source: PLoS One. 2013 Jan 14;8(1):e54507. doi: 10.1371/journal.pone.0054507 (PMC3544819; doi:10.1371/journal.pone.0054507)
Supplement: Table S2 — Summary of oligonucleotides used in this study. (PDF) [file pone.0054507.s006.pdf]

Supplemental Table S2.

Oligonucleotides

For qPCR, the following oligonucleotides were used:

qRT-PCR	Sequences (5' to 3')
Isl1	TCATCCGAGTGTGGTTTCAA
	TTCCTGTCATCCCCTGGATA
Mtmr2	TGGCCATGGAGATAAGAACC
	CAAATGCGGTAGGGAACTGT
Snca	GGCAGCTGGAAAGACAAAAG
	CCACTGCTCCTCCAACATTT
Gaa	GCTCCTACCCAGGTCCTTTC
	TCAGCAGTGAGACAGGGATG
Kif1b	ACACTGACATCGCTGACCTG
	AGAACGGCTGCTTGTCTCAT
Pcsk1n	ATTTTGGTGCTGCTGCTCTT
	AGTGCTCGTCTCAACCAAGG
Pax6	GCACATGCAAACACACATGA
	ACTTGGACGGGAACTGACAC
B2M	CATACGCCTGCAGAGTTAAGC
	GATGCTTGATCACATGTCTCG
Actb	5HEX/CCATACCC/ZEN/AGAAGGAAGGCTGGAA/3IABkFQ
	ATTGGCAACGAGCGGTT
	AGGTCTTTACGGATGTCAACG
Pax6	56-FAM/CTCGGGGAC/ZEN/CACTTCAACAGGAC/3IABkFQ
	GGCGGAGTTATGATACCTACAC
	GAACTTGGACGGGAACTGAC
Fat4	56-FAM/CCCATCGAG/ZEN/ACACTCAGACAGGTT/3IABkFQ
	CATTGAGGTGCTTTCCATGTC
	TCTCCACCATAGAGCACTGAG
Has2	56-FAM/ACATCTCCT/ZEN/CCAACACCTCCAACC/3IABkFQ
	CTTGACCCTGCCTCATCTG
	AAAGCCATCCAGTATCTCACG
Efnb2	56-FAM/TGATGCGAT/ZEN/CCCTGCGAATAAGGC/3IABkFQ
	TGTGAAGCCAAATCCAGGTT
	GATGATGACGATGAAGATGATGC
Trpm3	56-FAM/TCCGACCAT/ZEN/ACCAGACCATGTCCA/3IABkFQ
	GGAAGACCTGATTGGAAGAGATG
	GAGTGCATGCTATTGAGAACG

qRT-PCR	Sequences (5' to 3')
Nav1	AAACTCCCTCGACTCAGATCC
	TCTCGATCTGGTGACTCAATG
Actb	GCTGTATTC CCCTCCATCGTG
	CACGGTTGGCCTTAGGGTTCAG

qChIP	Sequences (5' to 3')
Isl1 (B)	GCCTCAGTACAGGGGAACAC
	CCCAAAAGTCCCCTATACCC
Isl1 (A)	AGAGCTGAAGGGGTCTCCA
	GCCAATTAGGCCATCTTCTG
Isl1 (NSR)	AGCAACGCTGGGATGTCTAT
	TTGCAAAAGAGAGCGAGTGA
Mtmr2 (A)	GGTCACCTGGAGATGAACAG
	GGTCACTTGGCTCCATTGTT
Mtmr2 NSR	CTCATCCTCAGCTTCCTTGG
	GCTATGCCCAAAACAGGAAA
Snca (A)	TGCAAAACTAAGCGGTACTGG
	CTGGCCAGACTGTGTGACTG
Snca (NSR)	TTCTGCCCACCACTAGCTCT
	TGGTGCTCAAGTGAGACTGC
Gaa (P)	GAGCTGGTGAAGGCTGTCTC
	CGATTCTCCAAAGCAGGAAC
Gaa (NSR)	TATCTAAAAGCCGGGCAGTG
	AGACCAGGCTGGAACTCAGA
Kif1b (P)	ATCCGGAGAGCTGGGATTAT
	GCTTCACCGTTTGAGCTGTT
Kif1b (NSR)	ATCGAGTCATGGGAGACACC
	TGAGATCCATCGAGTGTCCA
Pcsk1n (A)	AGGCTGTGCTGGAACTCACT
	GTGAAGCAAAGAGCCAGTCC
Pcsk1n (P)	CACAGCATGCAGCCAGTAAT
	GCTGAAATCAGGCTCTGGTC
Pcsk1n (NSR)	ATGGTGGCTTGGGTAAACTG
	GCTTCTTTCCCTGCAATGAC

For ISH, the following oligonucleotiedes were used:

ISH	Sequences (5' to 3')
Kif1b	CTGTTCGAGTGAGGCCCTTC
	CCCGTTCACTTCCTGCTAGA
Snca	GAAAGGACTTTCAAAGGCCA
	CAGCAACAAAAAGAAAACGATG

For EMSAs, the following oligonucleotiedes were used:

Loci	sites	Sequences (5' to 3')
Kif1b	1	ATGACCAATAGTAAAGCGTGGCTGGAAGAGGGG
		CCCCTCTTCCAGCCACGCTTTACTATTGGTCAT
	2	TAGGAAGCTTTCTGCTGGATACACTCATGCCTGAGCGTG
		CACGCTCAGGCATGAGTGTATCCAGCAGAAAGCTTCCTA
Snca	1	CTAGTGATTTATGCAACAGCATAGG
		CCTATGCTGTTGCATAAATCACTAG
	2	TATTGAAATAGTCTCTCTTTAAAATGG
		CCATTTTAAAGAGAGACTATTTCAATA
	3	GTTGGTTTTCTGCCTCTCAGAACTTTACCA
		TGGTAAAGTTCTGAGAGGCAGAAAACCAAC
	4	TCACCTATGCTGTTGCATAAATCACTAGTTTAT
		ATAAACTAGTGATTTATGCAACAGCATAGGTGA
